# Role of the cytopathologist during the procedure of fine-needle aspiration biopsy of thyroid nodules

**DOI:** 10.1186/s13244-021-01053-y

**Published:** 2021-08-09

**Authors:** F. Feletti, L. Mellini, F. Pironi, A. Carnevale, G. C. Parenti

**Affiliations:** 1 Department of Diagnostic Imaging Ausl Romagna, Unit of Radiology, S. Maria Delle Croci Hospital, Viale Randi 5, Ravenna, Italy; 2 DAMeTLab, Unit of Anatomical Pathology, S. M. Delle Croci Hospital, Ravenna, Italy; 3 Department of Radiology, University Radiology Unit, Sant’Anna University Hospital, Ferrara, Italy

**Keywords:** Health services administration, Patient care management, Intersectoral collaboration, Cost–benefit analysis, Interventional radiology

## Abstract

**Purpose:**

This study aimed to conduct a diagnostic and cost-effective analysis of the cytopathology assistance in the ultrasound (US)-guided fine-needle aspiration biopsy (FNAB) for characterising thyroid nodules.

**Materials and methods:**

We reviewed the reports relative to 9061 US-guided FNABs for the histologic definition of the nature of thyroid nodules: 45.4% completed with the cytopathologist assistance and 54.6% by the radiologist alone.

We also performed the cost-effectiveness analysis (CEA) of the procedure with and without the cytopathologist assistance.

**Results:**

We found a significant positive correlation between the adoption/non-adoption of cytopathologist assistance and the number of indeterminate (TIR1) (Chi-square; z-score, *Z* = 10.22; critical value 5%, *C* = 1.96; *p* < 0.001). The cytopathologist's absence was correlated with the number of TIR 1 (Pearson correlation, product–moment correlation *r* = 0.059; critical value 5%, C = 0.008; *p* < 0.001).

The total cost of the model's cytopathologist-assistance branch is 109.87€, while the total cost of the non-cytopathologist-assistance branch is 95.08€.

**Conclusion:**

The cytopathologist assistance resulted in fewer nondiagnostic results, thus excluding the procedure's repetition but involved a higher expense, mainly due to the professional cost of the pathologist's participation. These data may provide decision-makers in healthcare with a practical evidence based on the opportunity to include the cytopathologist assistance in the thyroid nodule's FNAB depending on the available resources and the population's expectance.

**Supplementary information:**

The online version contains supplementary material available at 10.1186/s13244-021-01053-y.

## Key points

- We examined a series of 9061 US-guided FNABs for thyroid nodules.
- We found that cytopathologist assistance significantly reduced the number of indeterminate results.
- The cytopathologist assistance increases the cost of the procedure.
- This study provides data of cost-effectiveness balance relative to an ordinary procedure.

## Introduction

Thyroid nodules have a high prevalence in healthy iodine-sufficient populations, reaching values up to 68% in studies based on ultrasound examination on randomly selected individuals [1–9]. Ultrasound (US)-guided fine-needle aspiration biopsy (FNAB) is the most accurate and cost-effective method for characterising thyroid nodules and for the selection of surgical candidates, with smear representing the method accepted to prepare the taken material [10–12]. While the diagnostic work-up of thyroid nodules through US-guided FNAB is justified to exclude thyroid cancer, it also represents an economic burden on healthcare systems [10]. The smear is prone to an inadequacy rate up to 34% [13], even in most experienced hands [10, 12], resulting in delayed diagnosis and higher costs for the healthcare system.

Many variables influence the diagnostic efficacy of US-guided FNAB; the technique of the specimen collection and evaluation and the preparation of the slide can play a decisive role [10]. In particular, the failure to aspirate a sufficient number of cells is crucial and, in turn, it may depend on the cystic or fibrotic structure of the aspirated nodule, by inexperienced aspirators, or the lack of on-site evaluation [14]. The on-site evaluation by cytopathologist is made in many medical centres and large community hospitals to decrease the nondiagnostic rate. Notwithstanding, the studies evaluating the true role of cytopathologist during the procedure of thyroid FNAB are sparse, and this practice's opportunity is still controversial [10, 15]. Cost-effectiveness analysis has never been performed [15, 16], and economic analyses are required to demonstrate the cost-effectiveness of the cytopathologist assistance [16]. Therefore, this study aimed to investigate the cost-effectiveness of cytopathologist assistance in the US-FNAB of the thyroid.

## Materials and methods

We retrospectively analysed the reports, relative to all the US-guided FNABs of thyroid nodules consecutively performed from the 1st January 2014 to the 31st December 2018 at our institution, with random adoption of the cytopathologist assistance.

Each examined report was relative to a specific FNAB procedure and had been filled in by the pathologist. The reports included the patient's demographic data (age, sex, acceptance date), the anatomic location of the examined nodule (right lobe, left lobe or isthmus), and whether cytopathology assistance was adopted. Each report also detailed the histologic findings according to the Consensus Statement AIT, AME, SIE and SIAPEC-IAP for the Classification and Reporting of Thyroid Cytology (Table 1) and the Bethesda System for Reporting Thyroid Cytopathology (BTC) [17, 18].

**Table 1:** Italian consensus statement AIT, AME, SIE and SIAPEC-IAP for the classification and reporting of thyroid cytology [16, 17]

Code	Diagnostic category	Risk of malignancy	Management
TIR 1	Non-diagnostic	Not determinated	Repeat US-guided FNAB
TIR 1C	Non-diagnostic cystic	Low risk	Evaluate clinical context and eventually repeat US-guided FNAB
TIR 2	Non-malignant (benign)	< 3%	Follow-up
TIR 3A	Low risk indeterminate	< 10%	Repeat FNAB/ Follow-up
TIR 3B	High risk indeterminate	15–30%	Surgical excision
TIR 4	Suspicion of malignancy	60–80%	Surgical excision with intraoperative examination
TIR 5	Malignant	95%	Surgical excision with diagnostic in-depth in selected cases (metastasis, lymphoma)

We examined all the reports relative to the needle aspiration of thyroid nodules. Exclusion criteria were the FNABs performed to refine a diagnosis of thyroiditis and FNABs performed on anatomic structures other than thyroids (e.g. parathyroid or lymph-nodes).

An overall number of three pathologists, seven radiologists and four nurses with specific expertise in these procedures were involved, in rotation, in these procedures.

The presence of cytopathologist assistance meant that a specially trained cytopathologist was present during the procedure. All on-site analysis was performed by the cytopathologist who had a multifaceted role. First, to decide, together with the radiologists, the best site of the nodule to perform the samples, based on ultrasonographic's features; then to aspire the cytologic material in a syringe connected with the needle placed by the radiologist.

Furthermore, the pathologist had to arrange the smear and to define whether the material in each sample was appropriate instantly. If the samples were inadequate, the pathologist had to suggest whether repeating the procedure on the same or another site.

In particular, when it was necessary to repeat the FNA at another site, the sampling was performed again within the same nodule of concern.

The pathologist generally proposed as a target the areas with the most vital aspects (e.g. tissue echostructure, any signals to the power Doppler) and avoiding components with colloidal or hemorrhagic appearance.

The sample was always assessed macroscopically and also microscopically whenever necessary.

The macroscopic evaluation leads to a judgement of inadequacy whether the smear had the typical consistency of pure blood; the material was scarce or not adequately distributed with the smear. A denser than blood or gelatinous consistency was generally considered macroscopically adequate and a partially haematic sample of dense consistency.

In case of scarce material of hard consistency, the primarily considered parameter for adequacy was the possibility to distribute the material in a thin layer on the slide.

Whenever required the cytopathologist also proceeded with the ROSE (Rapid On-Site Evaluation), which allows the on-site microscopic evaluation of the samples and involves the following steps: withdraw, swipe, colour, observe under the microscope, decide whether end the diagnostic procedure or continue [15].

When the cytopathologist was not present, the radiologist alone carried out the procedure, with the nurse's help. The radiologist also prepared the slide by sliding it into a standard way without any qualitative evaluation of the sample.

The data relative to all the reports included in the database relative to the thyroid nodules were exported on an Excel file.

Statistical analysis was carried with the software Wizard Pro V. 1.9.40., the Chi-square test and the Pearson correlation were used to test the null hypothesis that the cytopathologist assistance did not affect the number of the TIR 1 non-diagnostic results.

With our institution's quality control department's assistance, we calculated the cost of an FNAB without a cytopathologist assistance split in the cost for the radiological component (material costs + medical time) and the cost for the laboratory analysis of the material (Additional file 1).

The cost of the FNAB with cytopathologist assistance was also separately calculated.

For this purpose, a meantime for the execution of the FNAB of 20 min was estimated.

Utilising these data, we performed the cost-effectiveness analysis (CEA) of the procedure according to the available recommendation for cost-effectiveness in radiology and interventional radiology [19–21].

The software TreeAge Pro Healthcare was used to create the decision tree for a patient with a thyroid nodule.

## Results

### Demographics

During the considered period, our radiology service of our institution covered a geographical area of 1859.44 Km^2^, serving a population of 389, 456 (http://dati.istat.it/). Over 60 months (January 2014—December 2018), after removing ten duplications, our analysis reported the data relative to 6174 patients which they had undergone an overall number of 9061 US-guided FNABs for the histologic definition of the nature of thyroid nodules (Table 2).

**Table 2:** Breakdown of the obtained samples according to the classification and reporting of thyroid cytology for each procedure

TIR	Nodule 1			Nodule 2		Nodule 3	Nodule 4	Nodule 5	Nodule 6	Nodule 7	Tot
	1st FNAB	2nd FNAB	3rd FNAB	1st FNAB	2nd FNAB	1st FNAB	1st FNAB	1st FNAB	1st FNAB	1st FNAB	
1	466	17	0	31	1	0	0	0	0	0	515
2	4928	121	5	1712	6	400	107	33	10	2	7324
3	269	10	1	119	1	39	12	3	3	1	7839
4	50	3	1	20	0	8	0	0	1	0	83
5	206	8	0	75	3	28	3	1	0	0	324
6	157	2	0	18	0	7	4	0	1	0	407
7	98	3	1	52	0	11	1	1	0	1	168
Tot	6174	164	8	2027	11	493	127	38	15	4	9061

Specifically, the table describes that up to seven nodules were examined in each procedure. Concurrently, up to three repetitions of the procedure were carried out for each nodule to finalise the diagnosis. Each line reports the number of samples that fall under a label of the classification (TIR)

Most of the patients were females (74.3%; *n* = 4589), and the age range was 10–98 years (mean: 58 ± 0.3, IC: 95%). Each patient underwent one to seven FNABs on different nodules, and the data also included 183 repetitions for nodules classified as TIR1. The diagnosis was always obtained with a maximum of two repetitions of the procedure.

### Impact of cytopathologist assistance

A standardised FNAB procedure had been adopted using a 22-gauge needle and involving one to three passes, independently from the cytopathologist's presence.

The data relative to the result of each examination are reported in Table 2.

Out of 9061 procedures, 45.4% (*n* = 3988) were completed with the cytopathologist assistance and 54.6% (*n* = 5068) by the radiologist alone. The data were missing in five cases.

The presence/absence of cytopathologist assistance resulted in a correlation with the classification across the seven codes of the Classification and Reporting of Thyroid Cytology which showed a high significance (Chi-square test; X2 = 148.529, critical value 5%, C = 12.592; *p* < 0.001).

The indeterminate results (TIR 1) were 515 (5.7%). Among them, 22.3% (*n* = 115) were from specimens obtained with the cytopathologist assistance and 77.7% (*n* = 400) by the radiologist alone.

Overall, in our series, undetermined results (TIR 1) were 2.9% among the procedures carried out with the cytopathologist assistance and 7.9% among those carried out by the radiologist alone.

We found a significant positive correlation between the adoption/non-adoption of cytopathologist assistance and the number of indeterminate (TIR 1) (Chi-square; z-score, Z = 10.22; critical value 5%, C = 1.96; *p* < 0.001).

Moreover, the cytopathologist's absence was correlated with the number of TIR 1 (Pearson correlation, product–moment correlation *r* = 0.059; critical value 5%, C = 0.008; *p* < 0.001) making it possible to reject the null hypothesis.

### Cost analysis

The total cost of the thyroid nodule classification through US-guided FNAB was 87.77€ when the cytopathologist assistance was adopted, and 106.7 € when it was not.

**Fig. 1:**
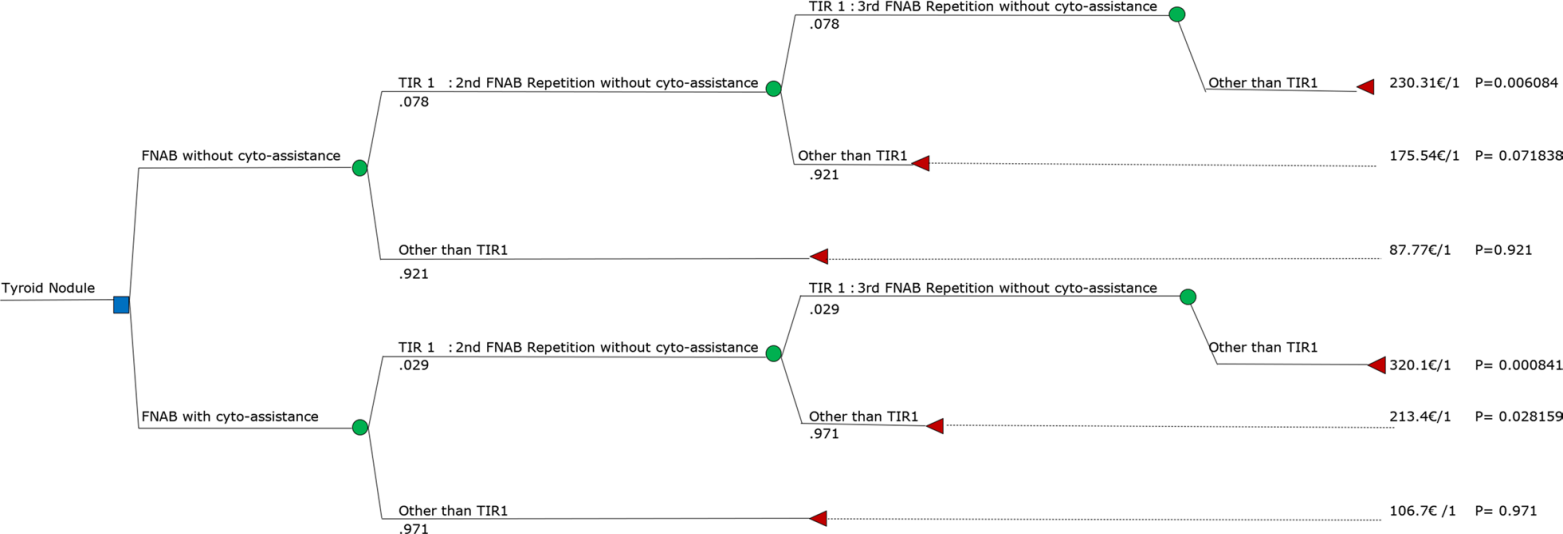
Decision tree relative to US-guided FNAB for characterising thyroid nodules

The definition of the CEA model is reported in Table. 3, while the decision tree is reported in Fig. 1.

**Table 3:** CEA model adopted for the comparative analysis of FNABs with and without cyto-assistance

Definition of the CEA model	
Reference case	Patient of age between 10 and 98 years old with a first US diagnosis of thyroid nodule
Strategies	Using cyto-assistance *vs* using FNABs without cyto-assistance. In both cases, non-diagnostic results TIR1 must be repeated until a final diagnosis is obtained
Time horizon	1 year
Perspective	Healthcare system perspective
Effective measure	Number of procedures to achieve a correct diagnosis
CEA model	Decision tree

The total cost of the model's cytopathologist-assistance branch is 109.87€, while the total cost of the non-cytopathologist-assistance branch is 95.08€.

## Discussion

Our series's indeterminate samples were in the lower zone of the range reported in the available literature, which describes a range between 1 and 34% [13, 14, 22].

The rate of nondiagnostic results (TIR 1) in a US-guided FNAB may depend on many variables. Among them are the number of passes, the negative pressure applied to the syringe, and sampling speed [10]. There is also a risk of contamination with blood or US transmission gel [10, 12].

Despite the importance of on-site evaluation of FNAB specimens in determining adequacy has been demonstrated in many fields [23–26], and even considered the gold standard and included in some guidelines [27, 28], the studies evaluating the role of cytopathologist during the thyroid FNAB are limited [15]. Scant cellularity and ineffective preparation techniques are common reasons of nondiagnostic procedures in up to 32% of FNABs in various organs, and thyroid included, rapid on-site evaluation by cytopathologist may be a useful tool for optimising the adequacy and quality of cytologic samples [29].

However, the cytopathologist's role during the procedure of US-guided FNAB is controversial, and some authors hypothesised that the execution of the procedure without cytopathology assistance is more cost-effective and more efficient in terms of workload and time [15].

Moreover, the FNAB without cytopathologist would be shorter, thus improving the patient comfort [15].

According to our results, the cytopathologist assistance can significantly improve the results of FNAB, because it reduces the number of nondiagnostic results.

In our experience, cytopathologist's presence generally allows reducing the number of multiple passes positively reflecting on the procedural time and patient discomfort.

By reducing the number of passes, the pathologist's presence may also reduce the risk of complications from the procedure, avoiding undersampling scenarios and allowing for the proper triage of materials.

In particular, to obtain qualitatively and quantitatively adequate preparations, smears should consist of only one cell layer; in thicker preparations, the key findings can be trapped between the layers and the preparation may assume a background colour, hindering the interpretation of the results. Moreover, fixation must be executed immediately to avoid cell degeneration, while incorrect fixation is a source of inaccurate diagnosis and affects the preparation's validity, leading to cell swelling and distortion of the cytoplasm.

An experienced pathologist can manage unexpected situations, such as obtaining scarce material of hard consistency, which may depend on very different conditions. Among them, the presence of old colloid, fibrotic nodules, calcific nodules, particularly aggressive nodules in which cells are closely cohesive and do not yield easily to the aspirate's pressure.

A pathologist can master the smearing technique and reduce the number of artefacts, therefore increasing the likelihood of accurate and definitive final diagnosis [29].

In conclusion, our analysis confirms that on-site cytopathology assistance effectively reduces the rate of nondiagnostic specimens, thus limiting the number of slices to be processed and examined [30–34].

According to our results, while the cytopathologist assistance may significantly reduce the unsatisfactory results of the procedure, thus improving the procedure's efficiency, it is more expensive.

However, according to examined data, the lack of cytopathologist assistance may lead every year to 90.5 TIR 1 results at our institution, leading to the repetition of the procedure, representing the 60.2% of the monthly capacity production capacity, covering the whole production of nearly three weeks.

On the contrary, based on a simulation, we estimated a run time for the pathology sample not exceeding three minutes. Since, in the meantime, the radiologist can perform other activities, including the monitoring of the sampling site in search of any complications (e.g. bleeding), we argued that cyto-assistance does not significantly modify the entire procedure's duration and, therefore, the size of a list.

While we were writing this piece, the outbreak of the coronavirus disease 2019 (Sars-COVID-19) has put the Italian health system in crisis, significantly lengthening the waiting lists for ordinary procedures, including FNAB. We believe that, in the future, any solutions that can reduce the people need to move, and the repetition of clinical investigations must be carefully considered. Simultaneously, the knowledge of cytopathologist assistance's real cost-effectiveness may guide the medical planners in deciding whether to include this procedure as part of routine US-guided FNAB workup of thyroid nodules.

A crucial point is the lack of a preliminary formal randomisation process relative to the patients' inclusion in the two groups, namely those who underwent the cytopathologist-assisted procedure and those treated by the radiologist alone.

However, the cytopathologists' participation in the FNABs procedures depended on their operative unit's organisation only, without any interference of the patients' demographic or clinical characteristics nor the nodules' ultrasound characteristics.

As a result, a de facto randomisation took place, allowing us to consider the inclusion to one of the two groups as a stochastic process.

Due to the retrospective nature of the study, we cannot exclude the existence of some bias.

Moreover, against 515 reported TIR1, we found only 183 repetitions of the procedure in our database. It is possible that after a nondiagnostic result, many patients had decided to move to other health facilities. However, this possibility probably does not significantly affect the results of our analysis.

Regardless, further research should be conducted prospectively to exclude the risk of the bias as previously discussed.

## Conclusion

In our series, the cytopathologist assistance during the FNAB for the cytologic diagnosis of thyroid nodules resulted in fewer nondiagnostic results, thus excluding the procedure's repetition, reducing the overall diagnostic time and the patient discomfort.

At the same time, however, it involved a higher expense, mainly due to the professional cost of the pathologist's participation.

Other variables may be crucial, including the risks of a delayed diagnosis in malignancy or the patients' psychological comfort that the present study did not consider.

These considerations leave space for a more in-depth research approach, also integrating these and different variables.

Notwithstanding the discussed limitations, the present study results provide data of cost-effectiveness balance related to an ordinary and frequent procedure.

These data may provide decision-makers in healthcare with a practical evidence base to decide whether to include the cytopathologist assistance in the thyroid nodule's FNAB depending on the available resources and the population's expectance.

## Supplementary information

**Additional file 1.** Breackdown of FNAB’s costs with and without the assistance of thecytopathologist.

## Data Availability

The datasets used and/or analysed during the current study are available from the corresponding author on reasonable request.

## Abbreviations

AIT: Thyroid Italian association
AME: Italian association of endocrinologists
CEA: Cost-effectiveness analysis
FNAB: Fine-needle aspiration biopsy
ROSE: Rapid on-site evaluation
SIAPEC-iap: Italian society of anatomo-pathology and cytopathology
Sie: Italian society of endocrinology
TBSRTC: Bethesda system for reporting thyroid cytopathology
US: Ultrasound
