# Supplementary material for: Role of the cytopathologist during the procedure of fine-needle aspiration biopsy of thyroid nodules
Source: Insights Imaging. 2021 Aug 9;12:111. doi: 10.1186/s13244-021-01053-y (PMC8350303; doi:10.1186/s13244-021-01053-y)
Supplement: Supplementary file 1 — Additional file 1. Breackdown of FNAB’s costs with and without the assistance of thecytopathologist. [file 13244_2021_1053_MOESM1_ESM.docx]

**06112 - BPS (PERC) (NEEDLE BPS) THYROID (ULTRASOUND)** WITH CYTOASSISTANCE

| **MATERIAL** | | | | |
| --- | --- | --- | --- | --- |
| **CODE** | **DESCRIPTION** | **UNITARY PRICE** | **AVERAGE CONSUMPTION (1 PROCEDURE)** | **COST PER PROCEDURE** |
| 0012043763 | NEEDLE ANEST SPIN_QUINCKE BPP_18GX90MM_ | 0.56 | 1.00 | 0.56 |
| 001234637 | GUIDE NEEDLE_ULTRAPRO II_14X61CM___ | 16.20 | 1.00 | 16.20 |
| 0012340052 | SHEET_FOLIODRAPE 2 STRATI_75X90CM | 0.44 | 1.00 | 0.44 |
| 001235146 | SIR LL 3/PZ S/NEEDLE_PLASTIPAK_20ML___ | 0.12 | 1.00 | 0.12 |
| 0012040695 | Gauze FOLD TNT ST__10X10CM 4STR____ | 0.01 | 1.00 | 0.01 |
| 001235205 | PROL BP_THREE STOP_15CM__PE_ | 0.51 | 1.00 | 0.51 |
| 0009100539 | BIO FIX BOTTLE 200 ML | 3.54 | 0.008 | 0.03 |
| 0011000685 | EFFE11 - SLIDES 76X26X1 WHITE | 0.10 | 1.00 | 0.10 |
| 0011000542 | FILM PER COVERSLIPPER_4770_70MT | 155.34 | 0.0007 | 0.11 |
| 0009110283 | ALCOHOLIC BLEND 95 ^ _DIAWHITE 95_ | 2.35 | 0.0007 | 0.00 |
| 0007104968 | XILENE CF 5 L | 14.03 | 0.001 | 0.02 |
| 0009110284 | COLORLESS ALCOHOLIC MIXTURE 99.8?_ | 2.40 | 0.001 | 0.00 |
| 0009110595 | PAPANICOLAOU'S SOLUTION 1B | 84.20 | 0.001 | 0.11 |
| 0009110594 | GIEMSA_SOLUTION BLU EOSINA _BLU | 80.52 | 0.001 | 0.11 |
| 0009110596 | PAPANICOLAOU SOLUTION 2A. ORANGE G | 63.21 | 0.001 | 0.08 |
| 0007000076 | FORMALDEHYDE 4% 500ML COD.1595 | 1.29 | 1.00 | 1.29 |
| 0011000752 | TUBE ST 50ML TRASP | 0.18 | 1.000 | 0.18 |
| 0009110282 | BIO AGAR_GEL FOR INCLUSIONS_ | 118.75 | 0.001 | 0.12 |
| 0007104901 | WAX ADDITIVE 56-58 C NO DMS0 | 5.70 | 0.001 | 0.01 |
| 0011000404 | BLADE MU MICROT_THERMO SCIENT_MX_35 | 1.16 | 1.000 | 1.16 |
| **dressing material** |  |  |  |  |
| 0012049377 | GLOVES NITRILE S/P_MEDIGUARD BLU _M_ | 0.03 | 6.00 | 0.19 |
| 0012046820 | GOWN CHIR ST_OPS ESSENTIAL_L__ST_ | 2.25 | 3.00 | 6.75 |
| 0012056205 | ROUND CAP W / ELASTIC __________ | 0.06 | 3.00 | 0.18 |
| 0012048776 | MASCH CHIR STD____C/LACCI MU_ | 0.02 | 3.00 | 0.07 |
| 0012202804 | SHOE COVER IN CPE___MU N/ST_ | 0.03 | 6.00 | 0.18 |
| **tot dressing material** |  |  |  | **7.37** |
| **SUBTOTAL** | | | | **28.53** |

| **PERSONNEL** | | | | |
| --- | --- | --- | --- | --- |
| **UNIT** | **QUALIFICATION** | **AVERAGE TIME (MIN)** | **AVERAGE COST per unit** | **TOTAL COST** |
| 1 | Radiologist | 20 | 0.94 | 18.77 |
| 1 | Nurse | 20 | 0.39 | 7.76 |
| 1 | Cytopathologist (reading) | 20 | 0.82 | 16.48 |
| 1 | Cytopathologist (material processing, cytoinclusion included) | 10 | 0.82 | 8.24 |
| 1 | Cytopathologist (on-site assessment) | 20 | 0.82 | 16.48 |
| **SUBTOTAL** | | | | **67.73** |

| **TECHNOLOGY** | | | | |
| --- | --- | --- | --- | --- |
| **EQUIPMENT** | **"ESTIMATE N PROCEDURES /year "** | **Value** | **Depreciation of machinery (per one year)** | **Unit value of the service** |
| **Ultrasound (cesp 606074)** | 6452 | 33550 | 6710.00 | 1.04 |
| **Automatic colourizer (cesp M000614799)** | 2000 | 94118 | 18823.60 | 9.41 |
| **SUBTOTAL** |  |  |  | **10.45** |

**TOTAL = 106.70**

**06112 - BPS (PERC) (NEEDLE BPS) THYROID (ULTRASOUND)** WITHOUT CYTOASSISTANCE

| **MATERIAL** | | | | |
| --- | --- | --- | --- | --- |
| **CODE** | **DESCRIPTION** | **UNITARY PRICE** | **AVERAGE CONSUMPTION (1 PROCEDURE)** | **COST PER PROCEDURE** |
| 0012043763 | NEEDLE ANEST SPIN_QUINCKE BPP_18GX90MM_ | 0.56 | 1.00 | 0.56 |
| 001234637 | GUIDE NEEDLE _ULTRAPRO II_14X61CM___ | 16.20 | 1.00 | 16.20 |
| 0012340052 | SHEET_FOLIODRAPE 2 STRATI_75X90CM | 0.44 | 1.00 | 0.44 |
| 001235146 | SIR LL 3/PZ S/NEEDLE _PLASTIPAK_20ML___ | 0.12 | 1.00 | 0.12 |
| 0012040695 | Gauze FOLD TNT ST__10X10CM 4STR____ | 0.01 | 1.00 | 0.01 |
| 001235205 | PROL BP_THREE STOP_15CM__PE_ | 0.51 | 1.00 | 0.51 |
| 0009100539 | BIO FIX BOTTLE 200 ML | 3.54 | 0.008 | 0.03 |
| 0011000685 | EFFE11 - SLIDES 76X26X1 WHITE | 0.10 | 1.00 | 0.10 |
| 0011000542 | FILM PER COVERSLIPPER_4770_70MT | 155.34 | 0.0007 | 0.11 |
| 0009110283 | ALCOHOLIC BLEND 95 ^ _DIAWHITE 95_ | 2.35 | 0.0007 | 0.00 |
| 0007104968 | XILENE CF 5 L | 14.03 | 0.001 | 0.02 |
| 0009110284 | COLORLESS ALCOHOLIC MIXTURE 998?_ | 2.40 | 0.001 | 0.00 |
| 0009110595 | PAPANICOLAOU'S SOLUTION 1B | 84.20 | 0.001 | 0.11 |
| 0009110594 | GIEMSA_SOLUTION BLU EOSINA _BLU | 80.52 | 0.001 | 0.11 |
| 0009110596 | PAPANICOLAOU SOLUTION 2A ORANGE G | 63.21 | 0.001 | 0.08 |
| 0007000076 | FORMALDEHYDE 4% 500ML COD1595 | 1.29 | 1.00 | 1.29 |
| 0011000752 | TUBE ST 50ML TRASP | 0.18 | 1.000 | 0.18 |
| 0009110282 | BIO AGAR_GEL FOR INCLUSIONS_ | 118.75 | 0.001 | 0.12 |
| 0007104901 | WAX ADDITIVE 56-58 C NO DMS0 | 5.70 | 0.001 | 0.01 |
| 0011000404 | BLADE MU MICROT_THERMO SCIENT_MX_35 | 1.16 | 1.000 | 1.16 |
| 0012049377 | GLOVES NITRILE S/P_MEDIGUARD BLU _M_ | 0.03 | 4.00 | 0.13 |
| 0012046820 | GOWN CHIR ST_OPS ESSENTIAL_L__ST_ | 2.25 | 2.00 | 4.50 |
| 0012056205 | ROUND CAP W / ELASTIC _________ | 0.06 | 2.00 | 0.12 |
| 0012048776 | MASCH CHIR STD____C/LACCI MU_ | 0.02 | 2.00 | 0.04 |
| 0012202804 | SHOE COVER IN CPE___MU N/ST_ | 0.03 | 4.00 | 0.12 |
| **SUBTOTAL** | | | | **26.07** |

| **PERSONNEL** | | | | |
| --- | --- | --- | --- | --- |
| **UNIT** | **QUALIFICATION** | **AVERAGE TIME (MIN)** | **AVERAGE COST per unit** | **TOTAL COST** |
| 1 | Radiologist | 20 | 0.94 | 18.77 |
| 1 | Nurse | 20 | 0.39 | 7.76 |
| 1 | Cytopathologist (reading) | 20 | 0.82 | 16.48 |
| 1 | Cytopathologist (material processing, cytoinclusion included) | 10 | 0.82 | 8.24 |
| **SUBTOTAL** | | | | **51.25** |

| **TECHNOLOGY** | | | | |
| --- | --- | --- | --- | --- |
| **EQUIPMENT** | **"ESTIMATE N PROCEDURES /year "** | **Value** | **Depreciation of machinery (per one year)** | **Unit value of the service** |
| **Ultrasound (cesp 606074)** | 6452 | 33550 | 6710.00 | 1.04 |
| **Automatic colourizer (cesp M000614799)** | 2000 | 94118 | 18823.60 | 9.41 |
| **SUBTOTAL** |  |  |  | **10.45** |

**TOTAL = 87.77**
